# *MEN1*/Menin regulates milk protein synthesis through mTOR signaling in mammary epithelial cells

**DOI:** 10.1038/s41598-017-06054-w

**Published:** 2017-07-14

**Authors:** Honghui Li, Xue Liu, Zhonghua Wang, Xueyan Lin, Zhengui Yan, Qiaoqiao Cao, Meng Zhao, Kerong Shi

**Affiliations:** Shandong Key Laboratory of Animal Bioengineering and Disease Prevention, College of Animal Science and Technology, Shandong Agricultural University, Taian, Shandong 271018 P. R. China

## Abstract

The *MEN1* gene, which encodes the protein Menin, was investigated for its regulatory role in milk protein synthesis in mammary glands. Menin responds to nutrient and hormone levels via the PI3K/Akt/mTOR pathway. Bovine mammary epithelial cells and tissues were used as experimental models in this study. The results revealed that the milk protein synthesis capacity of mammary epithelial cells could be regulated by *MEN1*/Menin. The overexpression of Menin caused significant suppression of factors involved in the mTOR pathway, as well as milk protein κ-casein (CSNK). In contrast, a significant increase in these factors and CSNK was observed upon *MEN1*/Menin knockdown. The repression of *MEN1*/Menin on the mTOR pathway was also observed in mammary gland tissues. Additionally, *MEN1*/Menin was found to elicit a negative response on prolactin (PRL) and/or insulin (INS), which caused a similar downstream impact on mTOR pathway factors and milk proteins. Collectively, our data indicate that *MEN1*/Menin could play a regulatory role in milk protein synthesis through mTOR signaling in the mammary gland by mediating the effects of hormones and nutrient status. The discovery of Menin’s role in mammary glands suggests Menin could be potential new target for the improvement of milk performance and adjustment of lactation period of dairy cows.

## Introduction

The *MEN1* gene, encoding the protein Menin which has 610 amino acid residues, was identified for its germline inactivating mutations, which cause the multiple endocrine neoplasia type 1 (MEN1) syndrome^1^. It is an autosomal dominant disorder characterized by tumors predominantly in the endocrine organs, including the parathyroid glands, pancreatic islets, and pituitary gland^1–3^. Thus, *MEN1*/Menin may play an indirect regulatory role in organism metabolism. For example, in the mammary gland, *MEN1*/Menin may function in the regulation of the endocrine hormone secretion of insulin (INS) or prolactin (PRL) on its target organs^1–3^.

In terms of its function in the mammary gland, Menin was found to directly interact with the estrogen receptor-α (ERα) in a hormone-dependent manner in breast cancer cells^4, 5^, thereby regulating the transcription of estrogen-responsive genes in breast cancer cells^4, 5^. Additionally, *MEN1* loss of heterozygosity caused inherited breast-ovarian cancer in humans^6^ and hyperplasia of the breast (female) and prostate (male) cells in mice^7, 8^. The above discoveries indicated that Menin plays an important regulatory role in mammary gland tissue. The objective of the study was to uncover the molecular mechanism of *MEN1*/Menin in regulating milk synthesis in the mammary glands.

Milk synthesis is the primary and important function of the mammary gland in mammals. Milk protein is an important component of milk and a source of nutrition for human consumption. Its yield is tightly regulated by nutritional and endocrine factors (hormones). The phosphatidylinositol-3-kinase (PI3K)/protein kinase B (PKB, also known as Akt)/mammalian target of rapamycin (mTOR) signaling pathway plays a central role in regulating cell proliferation, cell growth, apoptosis and metabolism by responding to the nutrient level and/or the circulating concentration of specific hormones^9, 10^. Recent studies have indicated that the regulation of protein translation through the PI3K/Akt/mTOR signaling pathway by nutrients, energy and hormones may be important in determining the milk protein production in mammary epithelial cells of dairy cows^11, 12^. The mTOR signaling cascade integrates nutritional and endocrine signals to alter the expression levels or phosphorylation status of eukaryotic initiation factor 4E (eIF4E)-binding protein 1 (4E-BP1), a translational repressor, and p70 ribosomal protein S6 kinase-1 (S6K1). The phosphorylation of S6K1 leads to the activation of several components of the protein translation apparatus in cells^13^.

Motivated by the search for the role of *MEN1* as a regulator in the milk protein synthesis that mediated by the mTOR signaling pathway in mammary glands, this study investigated the direct effects of *MEN1* in both the bovine mammary epithelial cell line MAC-T^14–16^ and bovine mammary gland tissues.

## Results

### *MEN1* knockdown promoted the expression of genes that regulate milk protein synthesis

**Figure 1:**
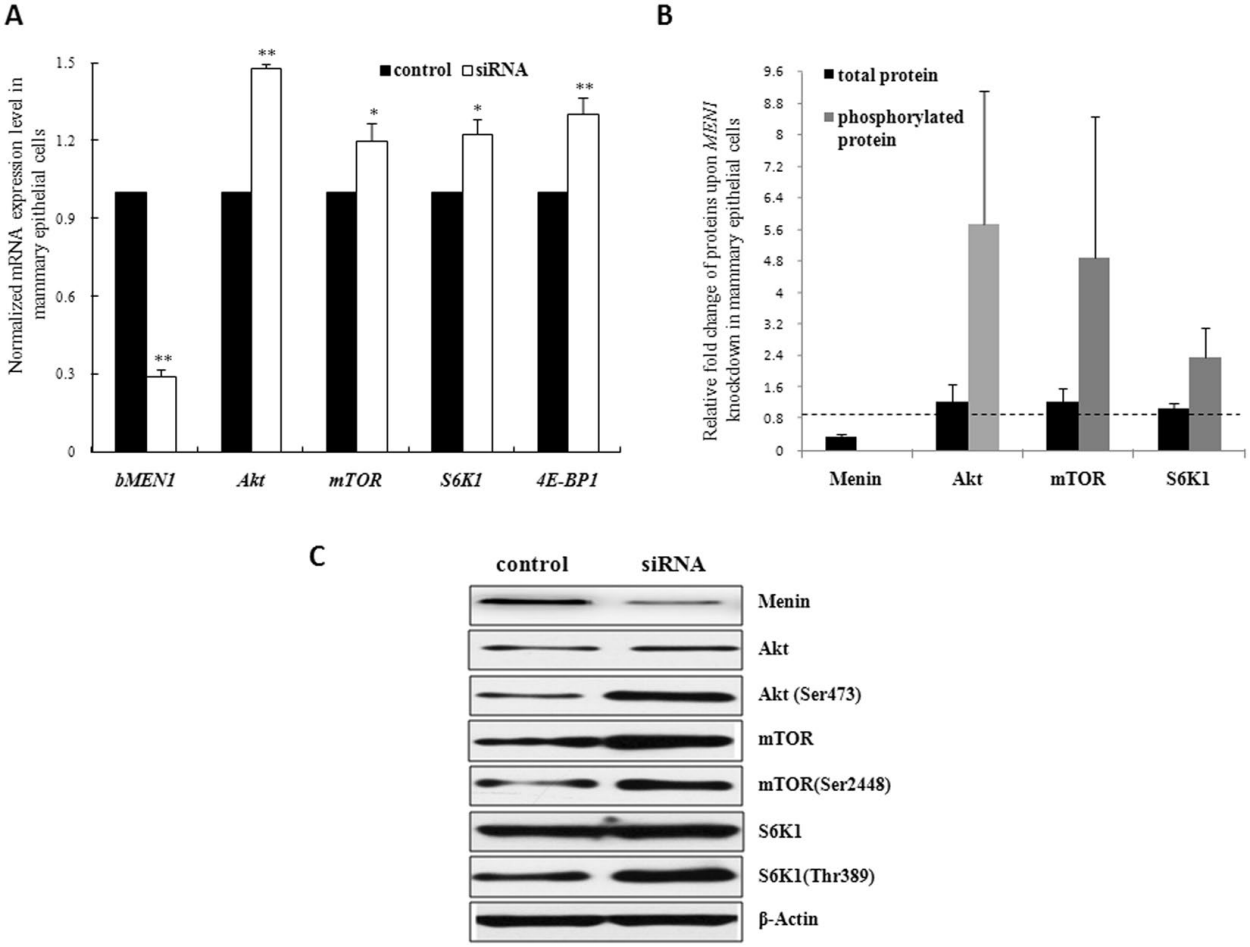
*MEN1* knockdown promoted the expression of genes that regulate milk protein synthesis in mammary epithelial cells. The expression levels of factors involved in the PI3K/Akt/mTOR pathway (Akt, mTOR, S6K1 and 4E-BP1) that are associated with milk protein synthesis were assessed at the mRNA ((**A**) n = 3) and total protein ((**B**) n = 3) levels by quantitative real-time PCR (qRT-PCR) and WB, respectively, in MAC-T cells at 24 h after transfection with a non-specific control (control) or a *MEN1* specific siRNA pool (siRNA). The phosphorylation levels of Akt at Ser473, mTOR at Ser2448, S6K1 at Thr389 and 4E-BP1 at Thr37/46 were detected simultaneously. The data are shown as the relative expression levels normalized to the internal control, β-actin. *P < 0.05, **P < 0.01. The horizontal dashed line represents the normalized level of their corresponding controls. Representative WB images of the expression of Menin, Akt, Akt phosphorylated at Ser473, mTOR, mTOR phosphorylated at Ser2448, S6K1 and S6K1 phosphorylated at Thr389 are shown in (**C**). PI3K, phosphatidylinositol-3-kinase; Akt, also known as protein kinase (**B**) Akt (Ser473), Akt phosplorylated at Ser473; mTOR, mammalian target of rapamycin; mTOR (Ser2448), mTOR phosplorylated at Ser2448; S6K1, p70 ribosomal protein S6 kinase-1; S6K1 (Thr389), S6K1 phosplorylated at Thr389; 4E-BP1, eukaryotic initiation factor 4E (eIF4E)-binding protein 1.

The factors involved in the PI3K/Akt/mTOR pathway have been known to participate in milk protein synthesis in the mammary gland^12, 13^. To assess the effect of *MEN1*/Menin overexpression, conversely, a knockdown system was applied in MAC-T cells (Supplementary Fig. S1). A 71.02% ± 0.38% knockdown efficiency for *MEN1* was observed at 24 h post-transfection. The expression levels of several components of the PI3K/Akt/mTOR pathway, *Akt*, *mTOR*, *S6K1* and *4E-BP1* were significantly increased at the mRNA level (Fig. 1A, *P* < 0.05). When their total and phosphorylated protein levels were detected, the protein Akt (phosphorylated at Ser473), mTOR (phosphorylated at Ser2448) and S6K1 (phosphorylated at Thr389) all showed increasing expression trend (Fig. 1B,C, *P* > 0.05), and it was markedly increased in some case (Fig. 1C). These results suggested that *MEN1* silencing could promote milk protein synthesis by upregulating the expression of factors involved in the mTOR signaling pathway.

### *MEN1* overexpression suppressed the expression of factors that regulate milk protein synthesis

**Figure 2:**
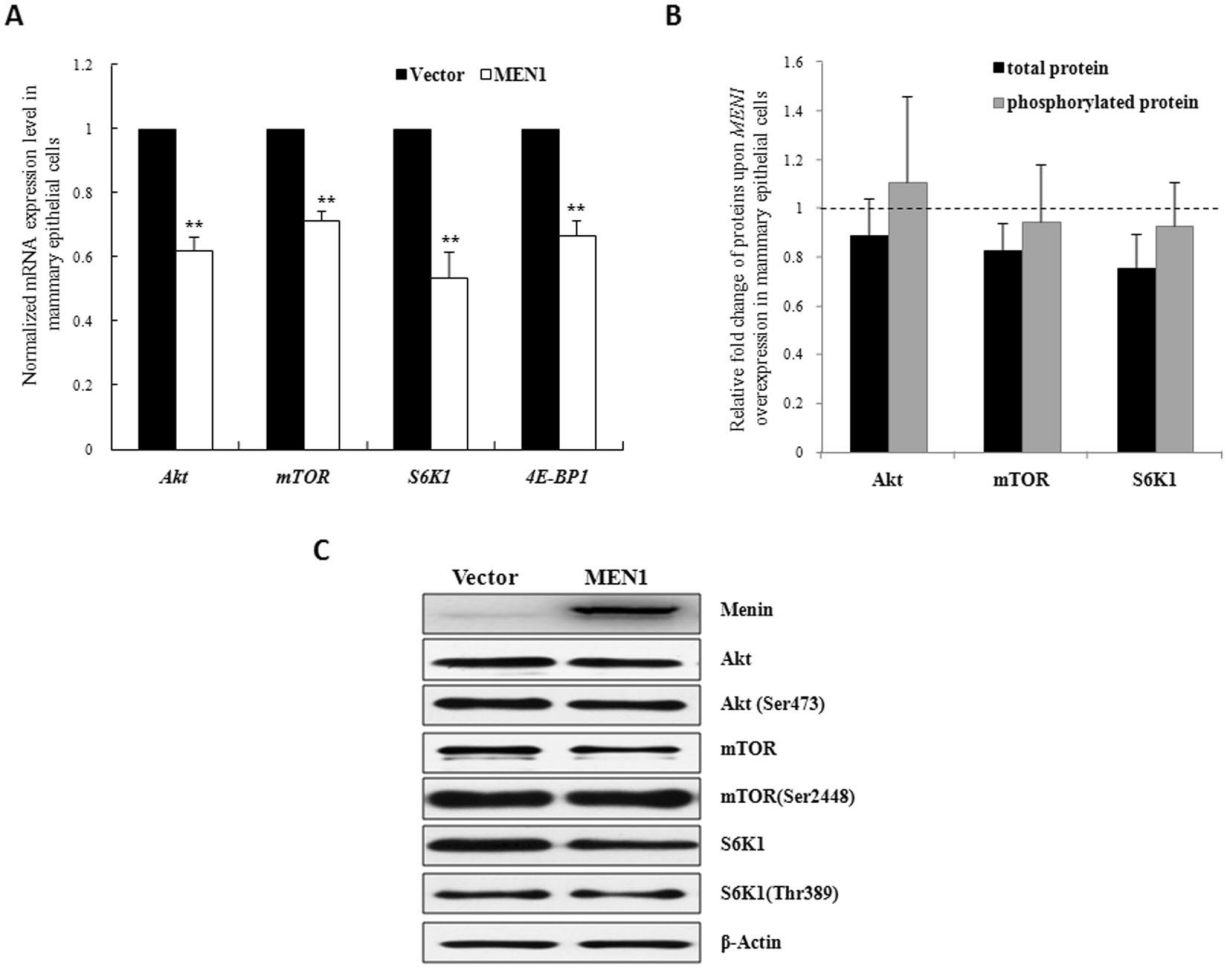
*MEN1* overexpression suppressed the expression of genes that regulate milk protein synthesis in mammary epithelial cells. The expression levels of factors involved in the PI3K/Akt/mTOR pathway (Akt, mTOR, S6K1 and 4E-BP1) that are associated with milk protein synthesis were assessed at the mRNA ((**A**) n = 3) and total protein ((**B**) n = 3) levels in MAC-T cells 24 h after transfection with the empty vector (Vector) or the *MEN1* expression plasmid (MEN1). The phosphorylation levels of Akt at Ser473, mTOR at Ser2448 and S6K1 at Thr389 were detected simultaneously. The immunoblotting detection of the total and phosphorylated 4E-BP1 at Thr37/46 using primary antibodies purchased from Abcam (Cat No. ab2606) and Cell Signaling Technology (Cat No. 2855) did not yield clear and specific bands (data not shown). The data are shown as the relative expression levels normalized to the internal control, β-actin. *P < 0.05, **P < 0.01. The horizontal dashed line represents the normalized level of their corresponding controls. Representative WB images of the expression of Menin, Akt, Akt phosphorylated at Ser473, mTOR, mTOR phosphorylated at Ser2448, S6K1 and S6K1 phosphorylated at Thr389 are shown in (**C**). PI3K, phosphatidylinositol-3-kinase; Akt, also known as protein kinase B; Akt (Ser473), Akt phosplorylated at Ser473; mTOR, mammalian target of rapamycin; mTOR (Ser2448), mTOR phosplorylated at Ser2448; S6K1, p70 ribosomal protein S6 kinase-1; S6K1 (Thr389), S6K1 phosplorylated at Thr389; 4E-BP1, eukaryotic initiation factor 4E (eIF4E)-binding protein 1.

To confirm the promotive effect of *MEN1*/Menin in mammary epithelial cells, MAC-T cells were transfected with either a plasmid encoding bovine Menin or an empty vector. By 24 h after transfection, the expression of Menin at both the mRNA and protein level was significantly increased (Supplementary Fig. S2, *P* < 0.01). The mRNA expression levels of *Akt*, *mTOR*, *S6K1* and *4E-PBP1*, were found to significantly decrease upon Menin overexpression compared to the cells transfected with the empty vector (Fig. 2A, *P* < 0.01). The total and phosphorylated protein measurements of Akt, mTOR and S6K1 also showed averaged lower expression level upon Menin overexpression compared to the corresponding negative controls (Fig. 2B,C, *P* > 0.05), except for the phosphorylation level of Akt. For example, as shown in Fig. 2C, the total protein of Akt, mTOR and S6K1 were all inhibited upon Menin overexpression. This indicated that Menin may downregulate milk protein synthesis by suppressing the expression of factors involved in the mTOR signaling pathway.

### *MEN1* may impact the expression of milk protein κ-casein

**Figure 3:**
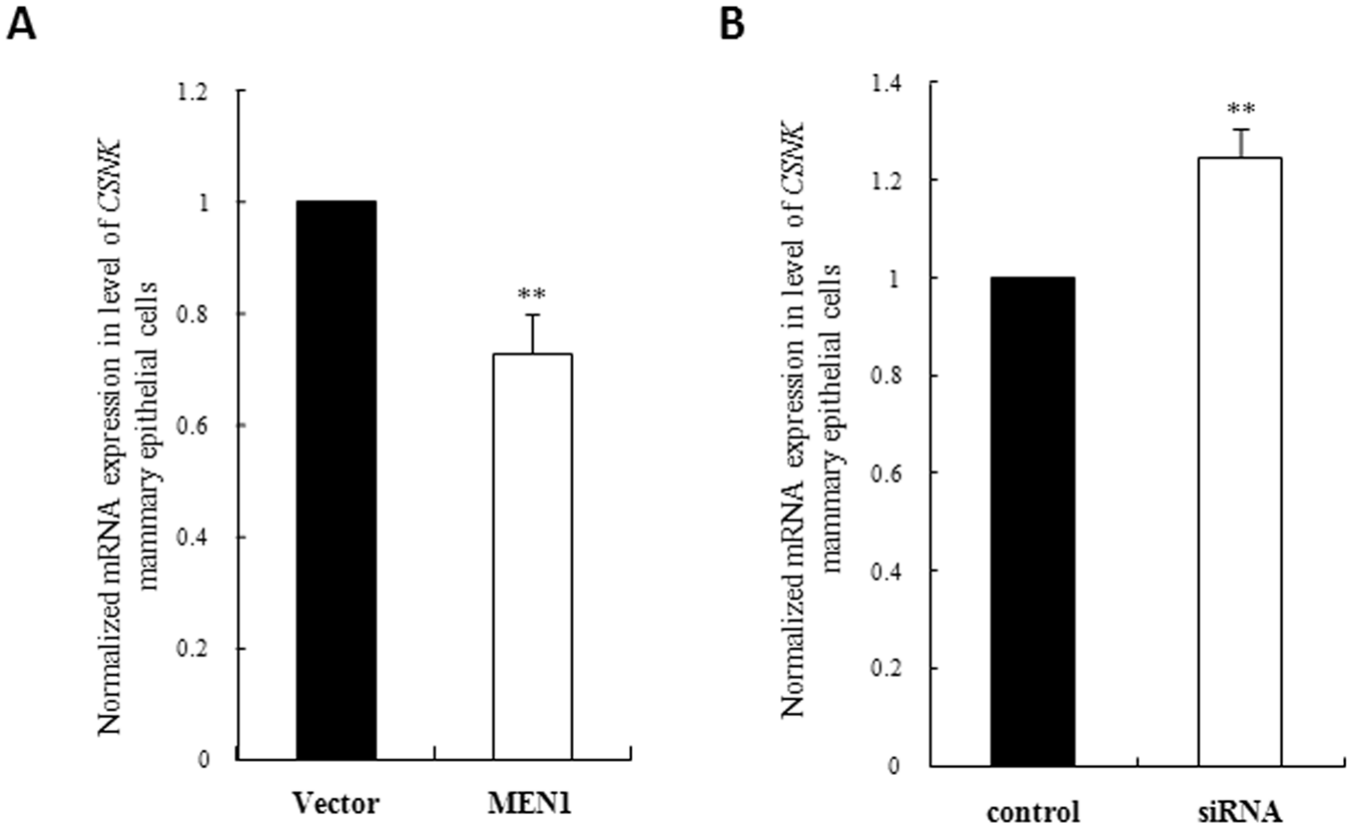
The RNA expression of *CSNK* (κ-casein) in mammary epithelial cells was downregulated upon *MEN1*/Menin overexpression and was upregulated upon *MEN1*/Menin knockdown. qRT-PCR was used to analyze the effect of *MEN1*/Menin on the expression of *CSNK* in MAC-T cells at 24 h after transfection with a *MEN1*-expressing plasmid (MEN1) or a *MEN1*-specific siRNA pool (siRNA) compared to their negative control cells transfected with the empty vector (Vector) or a non-specific control siRNA (control), respectively. The results, which are representative of three independent experiments, were used to determine statistical significance in both of the overexpression and knockdown system. *CSNK* was significantly suppressed upon transient *MEN1*/Menin overexpression (**A**), but it was significantly promoted (**B**) upon transient Menin knockdown. The data are shown as the relative expression levels normalized to the internal control, β-actin. *P < 0.05, **P < 0.01.

As the main components of the population of bovine milk proteins, acounting for approximately 80% of the total protein in bovine milk, the gene expression of the caseins, αs1-casein (*CSNAS1*), αs2-casein (*CSNAS2*), β-casein (*CSNB*) and κ-casein (*CSNK*), as well as β-lactoglobulin (*LGB)* and alpha-lactalbumin *(LALBA)*, were evaluated with *MEN1*/Menin over- and under-expression in MAC-T cells. *CSNK* was the only detectable casein, along with *LGB*, in MAC-T cells (Supplementary Fig. S4 and Table S1). The expression of *CSNK* was found to be significantly decreased upon transient Menin overexpression (Fig. 3A, *P* < 0.05), but it was significantly increased upon transient Menin knockdown (Fig. 3B, *P* < 0.01), compared to their corresponding negative controls. Therefore, these results suggested that *MEN1* could regulate milk protein synthesis by modulating the expression levels of the factors involved in mTOR signaling in MAC-T cells.

### In mammary gland tissues, modulated *MEN1* expression affected the expression of the genes regulating milk protein synthesis at different lactation periods

**Figure 4:**
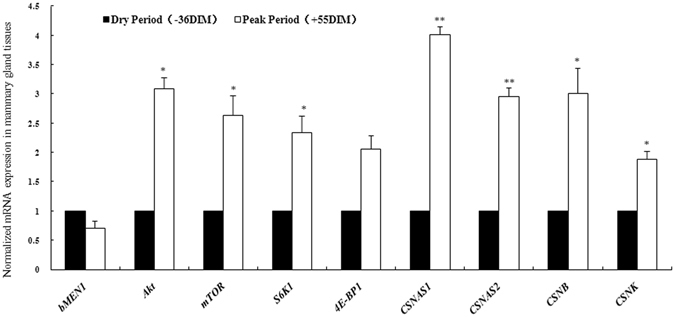
The effect of *MEN1/*Menin expression on the process of protein synthesis was confirmed at different lactation periods in mammary gland tissue. Bovine mammary gland tissues at two different lactation periods, peak milk period (+55DIM, days in milking) and dry period before parturition (-36DIM), were collected and used to detect the expression levels of *MEN1*, genes involved in the PI3K/Akt/mTOR pathway and the milk protein transcripts *CSNAS1* (αs1-casein), *CSNAS2* (αs2-casein), *CSNB* (β-casein), *CSNK* (κ-casein), *LGB* (β-lactoglobulin) and *LALBA* (alpha-lactalbumin). The expression of *MEN1* in the peak milk period (+55DIM) was lower (*P* = 0.06) than its expression in the dry period (-36DIM). The expression levels of *Akt*, *mTOR*, *S6K1* and *4E-BP1*, along with the 6 milk protein components, were increased in the peak milk period compared to those in the dry period. The results in mammary gland tissues were consistent with the observations from experiments conducted in the mammary epithelial cells.

To assess the physiological relevance of the observations for *MEN1* in the mammary epithelial cell line model, mammary gland tissues during two different lactation periods (Supplementary Table S2), the peak milk period and the dry period, were collected to detect the expression levels of *MEN1* and the genes involved in the PI3K/Akt/mTOR pathway. The expression of *Akt*, *mTOR*, *S6K1*, and *4E-BP1*, along with milk protein transcripts for *CSNAS1*, *CSNAS2*, *CSNB*, and *CSNK*, during the peak milk period (+55 days in milk, DIM) were increased compared with the dry period (-36DIM) (Fig. 4). Interestingly, the expression of *MEN1* in the peak milk period (+55DIM) showed lower mRNA level than that in the dry period (-36DIM) (Fig. 4, *P* = 0.06), so as its protein Menin expression level (Supplementary Fig. S4). These results in tissues were consistent with the observations in mammary epithelial cells, suggesting that *MEN1*/Menin at least partially modulates casein synthesis by negatively regulating the expression of the genes that are involved in the mTOR signaling pathway.

### *MEN1* could respond to the hormones, such as prolactin or insulin, therefore makes effect on milk protein synthesis in mammary epithelial cells

**Figure 5:**
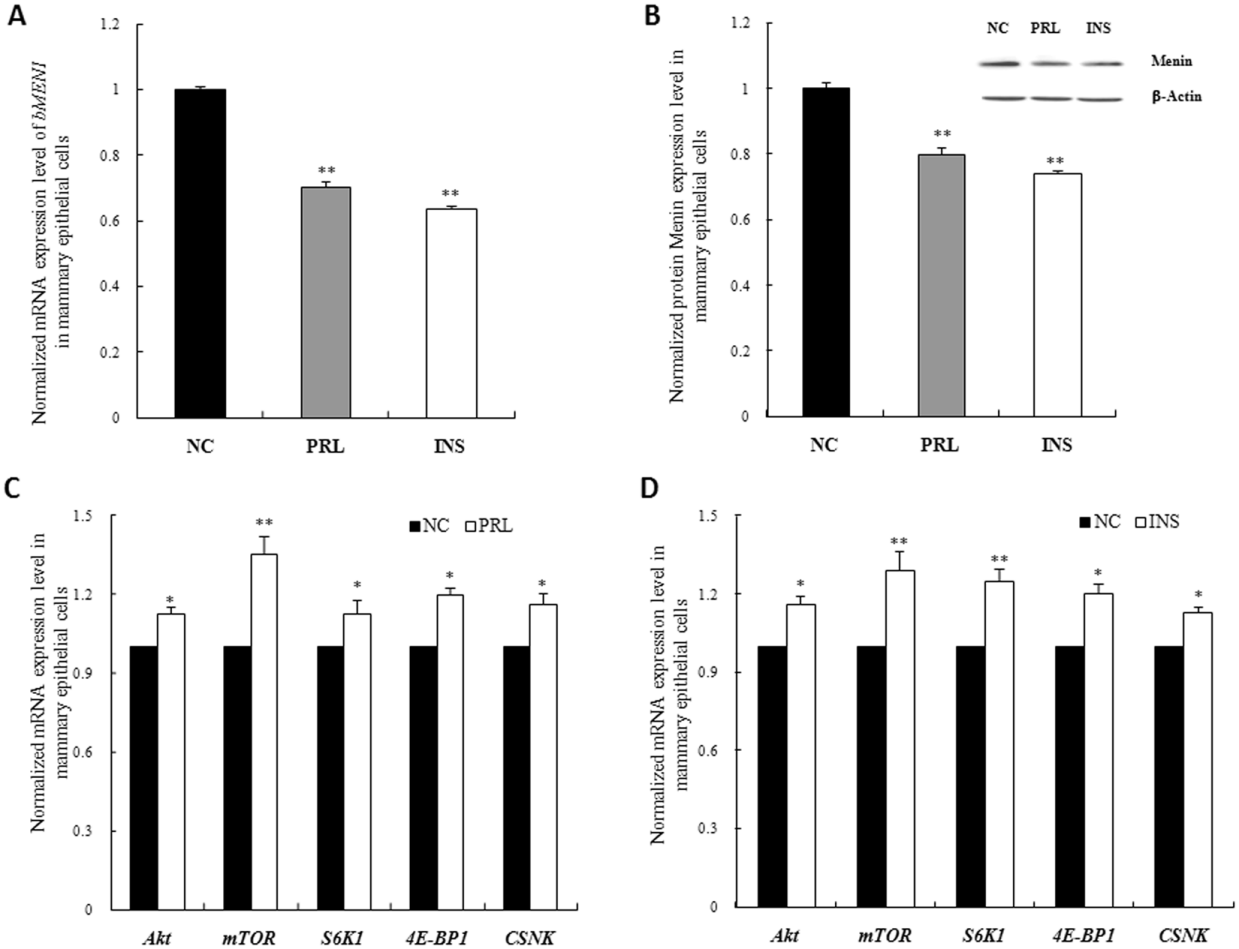
The endocrine hormones prolactin (PRL) and insulin (INS) modulated the expression of *MEN1*/Menin and its downstream factors involved in mTOR signaling. The *MEN1* mRNA (**A**) and Menin protein (**B**) level were significantly inhibited in MAC-T cells after 8 h of treatment with prolactin and insulin, compared to their saline-treated controls. The factors in the mTOR pathway, *Akt*, *mTOR*, *S6K1* and *4E-BP1*, along with *CSNK*, were found to be upregulated upon prolactin (**C**) and insulin (**D**) treatment. As shown above, the data are relative expression levels normalized to the internal control β-actin. *P < 0.05, **P < 0.01. Akt, also known as protein kinase (**B**) mTOR, mammalian target of rapamycin; S6K1, p70 ribosomal protein S6 kinase-1; 4E-BP1, eukaryotic initiation factor 4E (eIF4E)-binding protein 1; CSNK, κ-casein.

Mammary epithelial cells quickly respond to the environment of the body and sense nutrient and hormonal levels, enabling them to tightly control milk protein synthesis and secretion. To determine the upstream expression regulators of *MEN1* in mammary epithelial cells, the hormone prolactin (PRL) or insulin (INS) was used to treat MAC-T cells. Preliminary experiments were performed to obtain the optimal treatment concentration (Supplementary Fig. S5). The addition of prolactin or insulin to the culture of MAC-T cells markedly downregulated the expression of *MEN1* mRNA and its protein Menin (Fig. 5A,B, *P* < 0.01). Simultaneously, the genes involved in the mTOR pathway, *Akt*, *mTOR*, *S6K1* and *4E-BP1*, and as well as *CSNK*, were markedly upregulated upon treatment with prolactin or insulin (Fig. 5C). These results suggested that *MEN1*/Menin participates in the regulatory process of *CSNK* synthesis by prolactin or insulin through the mTOR signaling pathway.

## Discussion

Recent studies have suggested a possible regulatory role for *MEN1*/Menin in different tissues through the PI3K/Akt/mTOR signaling pathway^17–19^. Menin was found to directly interact with Akt1 and suppresses its kinase activity by regulating its translocation in the cytoplasm and/or plasma membrane^20, 21^. *MEN1* and the mTOR pathway genes were found to be frequently altered in pancreatic neuroendocrine tumors^22^. Menin might control insulin expression and/or secretion by modulating visinin-like protein 1 (VSNL1), which is a downstream target of the mTOR pathway^23^.

In mammary gland, it was suggested that *MEN1*/Menin played an important regulatory role in maintaining their normal function^4, 6–8^. In this study, we investigated how *MEN1*/Menin regulated the synthesis of milk protein in mammary epithelial cells. The bovine epithelial cell line MAC-T was used as a model for studying milk protein synthesis regulation^14–16^. The activation of the PI3K/Akt/mTOR pathway^12, 13, 24^ was revealed to be essential for the initiation of the transcription and translation of milk protein genes. Our results showed that the knockdown of *MEN1* gene caused the significant increase in the mRNA expression of main factors of the PI3K/Akt/mTOR signaling pathway, including Akt, mTOR, S6K1 and 4E-BP1. Furthermore, as shown in Fig. 1C, the total and phosphorylated protein of Akt, mTOR and S6K1 also increased upon the lower Menin expression in mammary epithelial cells^12, 13, 24^, although the up-regulation range of these proteins by Menin was relatively various. Conversely, the overexpression of the *MEN1* gene led to a significant reduction in the mRNA expression of these factors, so as the total and phosphorylated levels of mTOR and S6K1 upon increased Menin in some case (Fig. 2C). This led us to believe that *MEN1*/Menin could modulate the milk protein synthesis of mammary epithelial cells by inhibiting the PI3K/Akt/mTOR signaling pathway.

*In vivo*, we obtained similar results in the bovine mammary gland tissues. As expected, the RNA expressions of the factors involved in the PI3K/Akt/mTOR signaling pathway at peak milk stage were higher than that in the dry period stage, which was consistent with previous investigations in mammary tissues^24–26^. Interestingly, the expression of *MEN1*/Menin showed an opposite change at both the RNA and protein level. This further confirmed that *MEN1*/Menin negatively regulated the factors involved in the PI3K/Akt/mTOR signaling pathway in mammary epithelial cells, and this utimately had an effect on the synthesis of milk proteins, such as *CSNAS1*, *CSNAS2*, *CSNB*, *CSNK*, *LGB* and *LALBA*. Our data suggest that the lower expression level of *MEN1*/Menin at least partially causes of the higher milk protein yield at peak stage, yet we do not know whether other factors in addition to Menin play similar roles in milk protein synthesis.

Interestingly, *MEN1*/Menin was also found to negatively regulate STAT5 (signal transducer and activator of transcription 5) in both bovine mammary epithelial cells and bovine mammary tissues (Supplementary Fig. S6). STAT5 is one of the main factors in the JAK-STAT5 pathway, similar to the PI3K/Akt/mTOR pathway, which is another crucial signaling pathway that has been shown to regulate milk protein synthesis in mammary gland tissue^14, 27, 28^. With a lower level of *MEN1*/Menin at the peak milk stage in mammary gland tissues, the expression of *STAT5* was shown to be higher than that during the dry period. It was reported that induced a higher expression of STAT5 could increase the milk yield of dairy cow mammary epithelial cells^29^. This further indicated the important mediating role of Menin in milk protein synthesis in the mammary gland.

**Figure 6:**
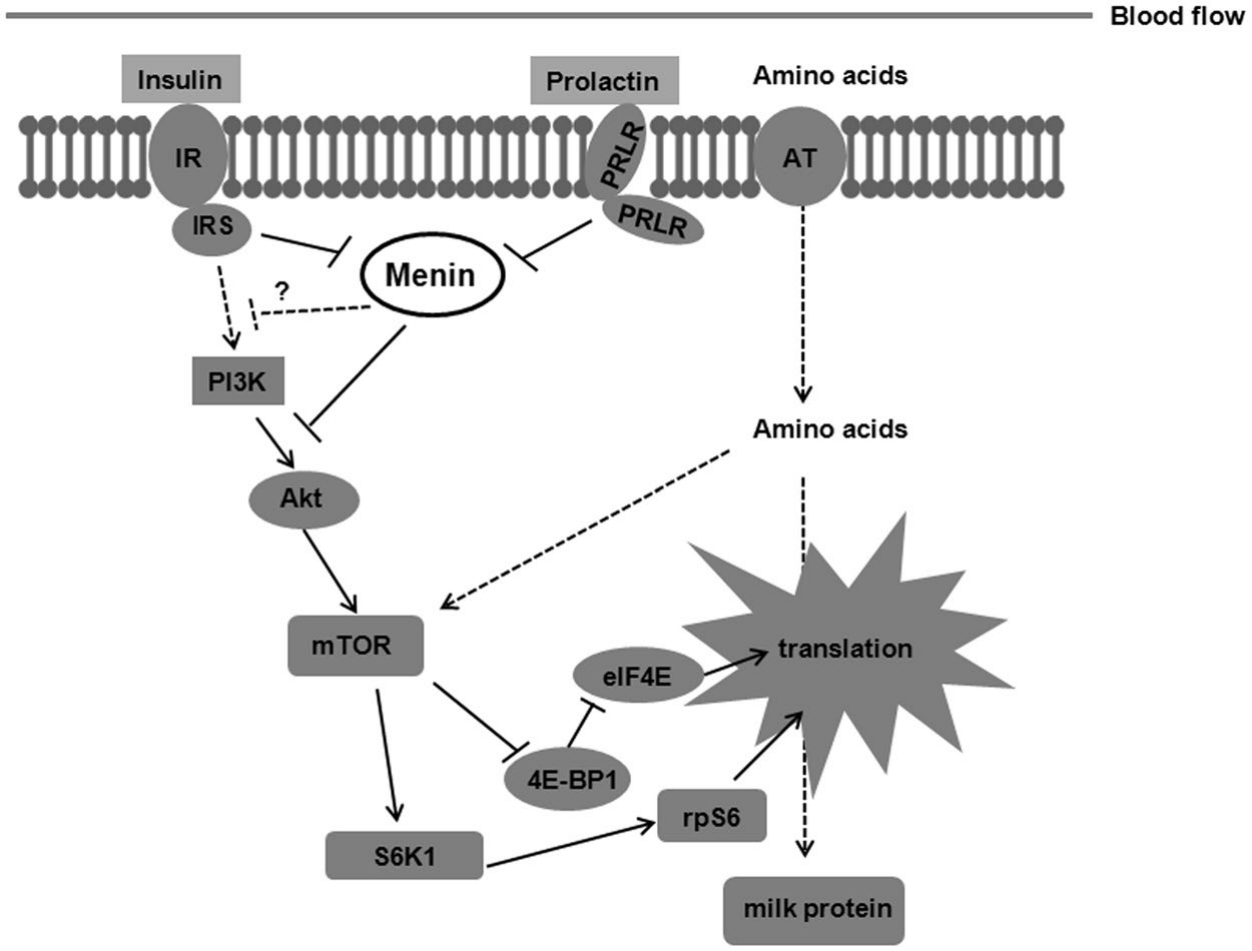
Mediation by Menin of milk protein synthesis through the PI3K/Akt/mTOR signaling pathway in mammary epithelial cells. Hormones such as insulin and prolactin that acts on epithelial cells modulate the *MEN1*/Menin expression level in cells, which further affects the abundance of the factors involved in mTOR signaling and eventually the synthesis of milk protein. Please see the text for further details. PI3K, phosphatidylinositol-3-kinase; Akt, also known as protein kinase (**B**) mTOR, mammalian target of rapamycin; IR, insulin receptor; IRS, IR substrate; PRLR, prolactin receptor; AT, amino acid transporter. The solid line represents fluxes; the dotted line represents possible effects or fluxes.

Milk protein, as one of the main components of milk, is *de novo* synthesized in the mammary epithelial cells by taking free amino acids through the amino acid transporter (AT) and small peptide molecules ingested from the blood as raw materials (Fig. 6). The synthesis process has been shown to be regulated by the levels of nutrition, energy and hormones near mammary epithelial cells^12, 30–33^. We found that *MEN1*/Menin in the mammary epithelial cells could respond to the level of prolactin or insulin, by inhibiting the expression of milk protein upon hormone treatment. In the bovine mammary gland tissue, possibly as a response to the secreted hormone (such as PRL), *MEN1*/Menin expression was modulated accordingly throughout the lactation period (Shi *et al*., unpublished data). For example, the expression level of Menin in the dry period tissue was lower than in the peak milking tissue. Thus, we showed that *MEN1*/Menin could negatively respond to the hormones and possible nutrients from diets in mammary glands on the process of milk protein synthesis through the PI3K/Akt/mTOR pathway (Fig. 6). Interestingly, Menin was found to respond to feeding and diet in the proximal duodenum, and it plays a negatively regulatory role on the glucose-dependent insulinotropic polypeptide (GIP) by inhibiting of the PI3K/Akt signaling pathway^19^.

This inhibition response mode of *MEN1*/Menin for the hormones in mammary glands is consistent with previous studies conducted in pancreatic islets of pregnant mice with prolactin^34^ and in mouse hepatocytes with insulin^35^. *MEN1*/Menin was originally discovered for its tumor suppression function in the pituitary (anterior lobe) and pancreatic islets^1–3^, which are well-known endocrine organs that secrete prolactin and insulin, respectively. Additonally, prolactin and/or insulin could be downstream factors of Menin, regulating their gene transcription^36, 37^. We hypothesize that there could be a negative feedback inhibition effect between *MEN1/*Menin and hormones in regulating milk protein synthesis in the mammary gland, and possibly other metabolic systems^19^.

Overall, *MEN1*/Menin can negatively modulate the milk protein synthesis capacity of mammary epithelial cells through the regulation of the PI3K/Akt/mTOR pathway (Fig. 6). Moreover, *MEN1*/Menin in the mammary epithelial cells was shown to regulate MAC-T cell growth and induce cell apoptosis when the expression of Menin was low (Shi *et al*., unpublished data). The downregulation of Menin expression at the peak milking stage might be one possible reason for the decrease in cell number, but increase in the protein synthesis capacity of mammary epithelial cells in the mammary gland^31^. This further supports the hypothesis that *MEN1*/Menin plays a regulatory role in the milk protein synthesis and/or milk yield by mediating the effects of hormones, possible nutrients and energy in the mammary gland. The results of our study might aid in the understanding of the function of Menin in the metabolism of mammary glands, and supply a new potential target improving the milk production performance of dairy cows. However, the molecular mechanism of how *MEN1/*Menin modulates the PI3K/Akt/mTOR signaling pathway needs to be further studied.

## Materials and Methods

### Cell culture

Immortalized bovine mammary epithelial cells (MAC-T) were grown in Dulbecco’s modified Eagle’s medium-F12 (DMEM/F12; Gibco, Waltham, MA) containing 10% fetal bovine serum (FBS; Biological Industries, Beth-HaEmek, Israel), 100 IU/mL penicillin, and 100 μg/ml streptomycin (Sigma, St. Louis, MO) at 37 °C under 5% CO_2_. Prior to the experimental treatments, the cells were detached with 0.25%-trypsin (Gibco) and transferred to a 6-well plate at a density of 5 × 10^5^ cells/well with antibiotic-free medium. For the hormone response experiment, MAC-T cells were treated using 5 μg/ml prolactin (PRL, Sigma) or insulin (INS,Sigma) for 8 h, which was followed by total RNA extraction and cell lysate production. Preliminary experiments were performed to optimize the treatment condition.

### Construction of the *MEN1* eukaryotic expression plasmid and transfection

The bovine *MEN1* (accession No. NM_001076161) eukaryotic expression plasmid pEGFP-C2-bMEN1 was constructed using the pEGFP-C2-Vector (Clontech, BD Biosciences) and PCR, with the bovine mammary gland tissue cDNA as the template and the specific primers (Forward: 5′-cccaagcttgccaccatggggctgaaggctgcccagaaaacg-3′; Reverse: 5′-cggaattctcagagacccttgcgctgccgcttgag-3; the restriction enzyme recognition site for cloning is underlined). MAC-T cells were transfected with 1.2 μg/well of pEGFP-C2-bMEN1 (bMEN1), as well as the negative control pEGFP-C2-Vector (vector), using the Lipofectamine^TM^ 2000 transfection reagent (Invitrogen, Carlsbad, CA). The total RNA and protein were isolated from the transfected cells 24 h post-transfection for further analysis. Time-course preliminary experiments were performed to optimize the efficiency of *MEN1*/Menin overexpression (Supplementary Fig. S1).

### Small interfering RNA transfection

Three bovine *MEN1*-specific siRNAs were designed and synthesized along with their negative controls (Ribobio, Guangzhou, China), targeting the sequences of 5′-ccgttgacctgtctctcta-3′, 5′-ccactgtcgtaaccgcaat-3′ and 5′-ccgagtgactacacgcttt-3′ in the *MEN1* gene. MAC-T cells were transfected with 100 nM of either a pool of *MEN1* siRNAs (siRNA) or negative control siRNA (control) using LF2000 (Invitrogen). Preliminary experiments were performed in order to obtain the highest knockdown efficiency (Supplementary Fig. S2).

### Quantitative real-time PCR (qRT-PCR)

The total RNA was isolated from homogenized bovine mammary gland tissues (10–15 mg) or cultured MAC-T cells using the RNAsimple Total RNA Kit (Tiangen, Beijing, China), and 1 μg RNA was reverse transcribed using the PrimeScript ^TM^ RT Reagent Kit with gDNA Eraser (Perfect Real Time) (TaKaRa, Dalian, China). The specificity of the PCR product was confirmed by melting curve analysis. All qRT-PCR assays were performed in triplicates in three independent experiments. The primer sequences are listed in Supplementary Table S1. The expression levels of the target mRNAs were normalized to the internal control β-actin mRNA for each transfection, and the 2^–ΔΔCT^ method was used to calculate the relative abundance of mRNA. The results are representative of at least three independent experiments to determine the statistical significance in both the overexpression and knockdown systems.

### Western blot analysis

MAC-T cellular lysate extracted after 24 h of transfection or mammary gland tissue lysate (appoximately 20 μg of total protein) was separated on a 10% SDS-PAGE gel and transferred onto nitrocellulose membranes using 200 mA of constant current. This was followed by a normal standard western blot (WB) protocol. The primary antibody against Menin (A300-105A, Bethyl, Texas, USA) was used at a 1:2000 dilution. The antibodies against the total and site-specific phosphorylated Akt (Ser473) (4691, Cell Signaling Technology, Beverly, MA, USA; AF0908, Affinity Biosciences, Cincinnati, OH, USA, respectively), mTOR (Ser2448) (2983, Cell Signaling Technology, Beverly, MA, USA and ab109268, Abcam, Cambridge, MA, USA, respectively), S6K1 (Thr389) (ab32359, Abcam, Cambridge, MA, USA and 9234, Cell Signaling Technology, Beverly, MA, USA, respectively), and β-actin (AA128, Beyotime, Jiangsu, China) were used at a 1:1000 dilution. β-actin was used as the loading control. The HRP-conjugated secondary antibody (Beyotime, Jiangsu, China) was diluted by 1000 as the working solution. Chemiluminescence detection was performed using BeyoECL Plus (Beyotime, Beijing, China). The immunoblotting detection of the total and phosphorylated 4E-BP1 at Thr37/46 using primary antibodies purchased from Abcam (Cat No. ab2606) and Cell Signaling Technology (Cat No. 2855) did not yield clear and specific bands (data not shown). The data are shown as the relative expression level normalized to their corresponding negative controls. The results are representative of three independent experiments that were used to determine the statistical significance.

### Ethics statement

All experiments were carried out according to the Regulations for the Administration of Affairs Concerning Experimental Animals published by the Ministry of Science and Technology (China, 2004) and approved by the Animal Care and Use Committee of Shandong Agricultural University (Shandong, China).

### Mammary gland tissue collection

Mammary gland tissues from six healthy Holstein cows, with an average weight of 602 ± 19.2 kg, were collected at the Holstein Cattle Association Jiabao Farm in Shandong Province. The randomly selected rear mammary gland was sanitized, and a subcutaneous injection of 1% procaine was administered after the animal was bundled. Next, the skin was cut with an incision 0.5–1 cm in length, and the subcutaneous tissue was peeled off until the mammary gland tissue was exposed. Mammary tissues were biopsied using the Bard Magnum biopsy system (Bard Peripheral Vascular, Inc., Tempe, AZ, US) followed by surgical suturing of the skin. The animal was administered ketaprofen and penicillin G procaine by intravenous injection immediately after biopsy. The same injection protocol was performed for the next 3–5 days, and the animal was monitored for up to two weeks depending on its condition. Mammary tissue samples were immediately snap-frozen in liquid nitrogen and stored at −80 °C for later analysis. Tissue samples were collected on the day that corresponded with the peak milk period at +55DIM (55 ± 4.3 DIM, n = 3) and the dry period before parturition at −36DIM (−36 ± 2.8, n = 3). The milk production information for these cows is shown in Supplementary Table S2. Cows were housed in a free stall barn with constant access to water and feed. Diets were formulated to meet all NRC (2001) recommendations for mid-lactation and dry cows using the Cornell-Penn-Miner system (CPM-Dairy, version 3.0.7). Feed was provided *ad libitum*, and the cows were milked three times daily.

### Statistical analysis

All the data were expressed as the mean ± S.E.M of at least three independent experiments or animals. The data statistics was analyzed using a *t*-test in the SAS 8.2 software (SAS Institute Inc., Cary, NC, USA). For each statistics, data sets obtained from at least three different biological repeats were used for analysis. The data for each biological repeat would make an individual difference. As for the qRT-PCR results, the individual difference would come from three pairs of CT values corresponding to three pairs of technical replicate reactions. Statistical differences were considered statistically significant if the *P* values were < 0.05 (*) or *P* < 0.01 (**).

## Electronic supplementary material

supplementary Figures and Tables

## References

[1] Chandrasekharappa SC (1997). Positional cloning of the gene for multiple endocrine neoplasia-type 1. Science.

[2] Schussheim DH (2001). Multiple endocrine neoplasia type 1: new clinical and basic findings. Trends in Endocrinology and Metabolism.

[3] Agarwal SK (2005). Menin molecular interactions: insights into normal functions and tumorigenesis. Hormone and Metabolic Research.

[4] Dreijerink KM (2006). Menin links estrogen receptor activiation to histone H3K4 trimethylation. Cancer research.

[5] Imachi H (2010). Menin, a product of the MENI gene, binds to estrogen receptor to enhance its activity in breast cancer cells: possibility of a novel predictive factor for tamoxifen resistance. Breast Cancer Research Treatment.

[6] Papi L (2009). Germline mutations in MEN1 and BRCA1 genes in a woman with familial multiple endocrine neoplasia type 1 and inherited breast-ovarian cancer syndromes: a case report. Cancer Genetics and Cytogenetics.

[7] Seigne C (2010). Characterisation of prostate cancer lesions in heterozygous Men1 mutant mice. BMC Cancer.

[8] Seigne C (2013). High incidence of mammary intraepithelial neoplasia development in Men1-disrupted murine mammary glands. Journal of Pathology.

[9] Hara K (1998). Amino acid sufficiency and mTOR regulate p70 S6 kinase and eIF-4EBP1 through a common effector mechanism. Journal of Biological Chemistry.

[10] O’Neil TK, Duffy LR, Frey JW, Hornberger TA (2009). The role of phosphoinositide 3-kinase and phosphatidic acid in the regulation of mTOR following eccentric contractions. Journal of Physiology.

[11] Yang X (2008). The mammalian target of rapamycin-signaling pathway in regulating metabolism and growth. Journal of Animal Science.

[12] Burgos SA, Dai M, Cant JP (2010). Nutrient availability and lactogenic hormones regulate mammary protein synthesis through the mammalian target of rapamycin signaling pathway. Journal of Dairy Science.

[13] Hayashi AA (2009). Initiation and elongation steps of mRNA translation are involved in the increase in milk protein yield caused by growth hormone administration during lactation. Journal of Dairy Science.

[14] Zhou Y, Akers RM, Jiang H (2008). Growth hormone can induce expression of four major milk protein genes in transfected MAC-T cells. Journal of Dairy Science.

[15] Hosseini A, Sharma R, Bionaz M, Loor JJ (2013). Transcriptomics comparisons of Mac-T cells versus mammary tissue during late pregnancy and peak lactation. Advances in dairy research.

[16] Suzuki Y (2015). Chemerin is a novel regulator of lactogenesis in bovine mammary epithelial cells. Biochemical and Biophysical Research Communications.

[17] Wang X, Proud CG (2006). The mTOR pathway in the control of protein synthesis. Physiology (Bethesda).

[18] Ehehalt F, Franke E, Pilarsky C, Grutzmann R (2010). Molecular pathogenesis of pancreatic neuroendocrine tumors. Cancers (Basel).

[19] Angevine KR (2012). Menin and GIP are inversely regulated by food intake and diet via PI3K/AKT signaling in the proximal duodenum. Nutrition and Diabetes.

[20] Wang Y, Ozawa A, Zaman S (2011). The tumor suppressor protein Menin inhibits AKT activation by regulating its cellular localization. Cancer Research.

[21] Hughes E, Huang C (2011). Participation of Akt, menin, and p21 in pregnancy-induced beta-cell proliferation. Endocrinology.

[22] Jiao Y, Shi C, Edil BH (2011). DAXX/ATRX, MEN1, and mTOR pathway genes are frequently altered in pancreatic neuroendocrine tumors. Science.

[23] Shi KR, Parekh VI, Swarnava R, Desai SS, Agarwal SK (2013). The embryonic transcription 1 factor Hlxb9 is a Menin interacting partner that controls pancreatic β-cell proliferation and the expression of insulin regulators. Endocrine-Related Cancer.

[24] Toerien CA, Trout DR, Cant JP (2010). Nutritional stimulation of milk protein yield of cows is associated with changes in phosphorylation of mammary eukaryotic initiation factor 2 and ribosomal s6 kinase 1. Journal of Nutrition.

[25] Bionaz M, Loor JJ (2011). Gene network driving bovine mammary protein synthesis during the lactation cycle. Bioinformatics and Biology Insights.

[26] Shi H (2015). Genes regulating lipid and protein metabolism are highly expressed in mammary gland of lactating dairy goats. Functional & Integrative Genomics.

[27] Akers RM (1990). Lactation physiology: a ruminant animal perspective. Protoplasma.

[28] Yang J, Kennelly JJ, Baracos VE (2000). The activity of transcription factor Stat5 responds to prolactin, growth hormone, and IGF-1 in rat and bovine mammary explant culture. Journal of Animal Science..

[29] Liu XF (2012). Stat5a increases lactation of dairy cow mammary gland epithelial cells cultured *in vitro*. In Vitro Cellular & Developmental Biology-Animal.

[30] Rius AG (2010). Regulation of protein synthesis in mammary glands of lactating dairy cows by starch and amino acids. Journal of Dairy Science.

[31] Capuco AV, Wood DL, Baldwin R, Mcleod K, Paape MJ (2001). Mammary cell number, proliferation, and apoptosis during a bovine lactation: relation to milk production and effect of bST. Journal of Dairy Science.

[32] Appuhamy JADRN (2014). Effects of AMP-activated protein kinase (AMPK) signaling and essential amino acids on mammalian target of rapamycin (mTOR) signaling and protein synthesis rates in mammary cells. Journal of Dairy Science.

[33] Arriola Apelo SI (2014). Isoleucine, leucine, methionine, and threonline effects on mammalian target of rapamycin signaling in mammary tissue. Journal of Dairy Science.

[34] Karnik SK (2007). Menin controls growth of pancreatic beta-cells in pregnant mice and promotes gestational diabetes mellitus. Science.

[35] Wuescher L (2011). Insulin regulates menin expression, cytoplasmic localization, and interaction with FOXO1. American. Journal of Physioloy. Endocrinology and Metabolism.

[36] Namihira H (2002). The multiple endocrine neoplasia type 1 gene product, Menin, inhibits the human prolactin promoter activity. Journal of Molecular Endocrinology.

[37] Sayo Y (2002). The multiple endocrine neoplasia type1 gene product, menin, inhibits insulin production in rat insulinoma cells. Endocrinology.

