# Supplementary material for: MEN1/Menin regulates milk protein synthesis through mTOR signaling in mammary epithelial cells
Source: Sci Rep. 2017 Jul 14;7:5479. doi: 10.1038/s41598-017-06054-w (PMC5511157; doi:10.1038/s41598-017-06054-w)
Supplement: Supplementary file 1 — supplementary Figures and Tables [file 41598_2017_6054_MOESM1_ESM.doc]

***MEN1*/menin regulates milk protein synthesis through mTOR signaling in mammary epithelial cells**

Honghui Li, Xue Liu, Zhonghua Wang, Xueyan Lin, Zhengui Yan, Qiaoqiao Cao, Meng Zhao, Kerong Shi ^*^

**
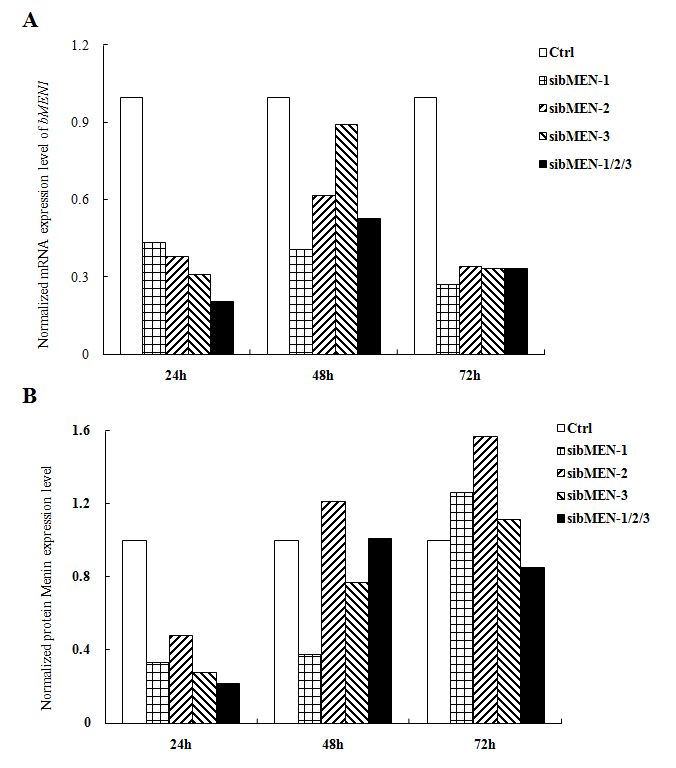
**

**Supplementary Figure S1. Optimization of the Menin knockdown. MAC-T cells were transfected with each of three *MEN1*-specific siRNAs (sibMEN1-1, -2, or -3) in separate and/or in combination (sibMEN-1/2/3), respectively. The expression level of *MEN1* mRNA (A) and Menin protein (B) were determined by qRT-PCR and western blot at 24 h, 48 h and 72 h after transfection. The data are shown as relative expression levels normalized to an internal control β-Actin.**

**
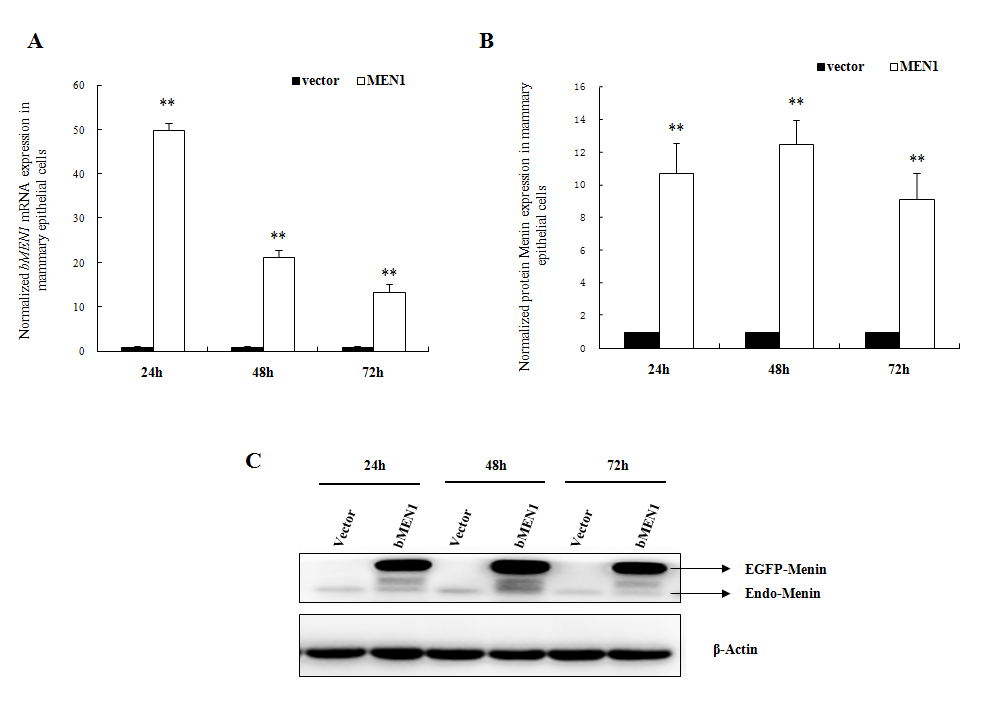
**

**Supplementary Figure S2. Optimization of the Menin over-expression. (A) The expression level of *MEN1* mRNA (A) and Menin protein (B) as determined by qRT-PCR and western blot in MAC-T cells at 24 h, 48 h and 72 h after transfection with the empty vector (Vector) or the *MEN1* expression plasmid (MEN1). The data are shown as relative expression levels normalized to an internal control β-Actin. **P*<0.05, ** *P*<0.01. Representative WB images of the Menin expression are shown in (C).**

**
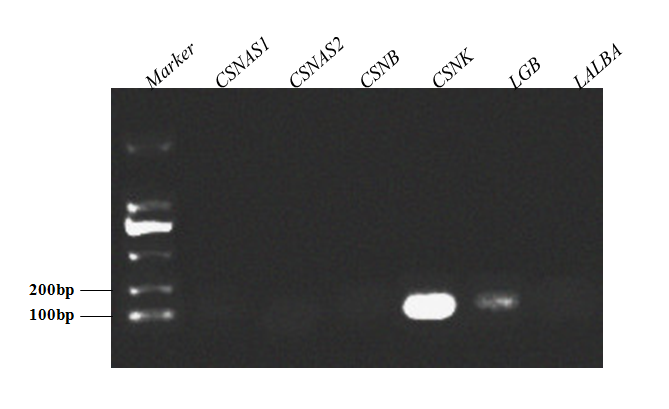
**

**Supplementary Figure S3. The expression of milk protein key components κ-casein (*CSNK*) and β-lactoglobulin (*LGB*) can be detected in MAC-T cells. The primer pairs target to *CSNK* and *LGB* in previously published articles (Zhou *et al.* 2008; Suzuki *et al.* 2015) were referred for gene expression detection. *CSNAS1,* αs1-casein; *CSNAS2 ,* αs2- casein; *CSNB,* β-casein; *CSNK,* κ- casein; *LGB,* β-lactoglobulin*; LALBA,* alpha-lactalbumin.**

**
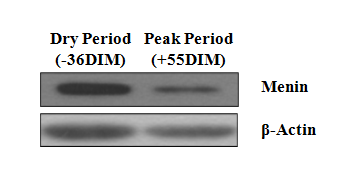
**

**Supplementary Figure S4. The Menin protein expression at the dry period stage (-36DIM) of mammary gland tissue was lower than that at the peak milk stage (+55DIM). Representative western blot images of Menin expression in bovine mammary gland tissues were shown.**

**
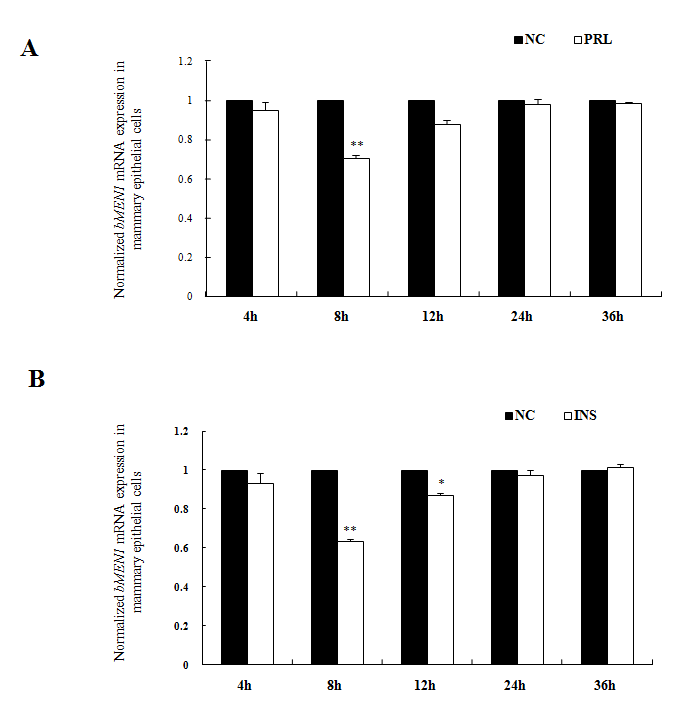
**

**Supplementary Figure S5. The *MEN1* expression in MAC-T cells was significantly suppressed at 8 h after treatment with prolactin (PRL) (A) and insulin (INS) (B), compared to saline-treated negative control cells, respectively. Bovine mammary epithelial cells MAC-T cells were treated with 5 μg/mL PRL or INS, *MEN1* mRNA expression was then detected at 4 h, 8 h, 12 h, 24 h and 36 h, respectively. The data are shown as relative expression levels normalized to an internal control β-Actin. * *P <* 0.05, ** *P <* 0.01.**

**
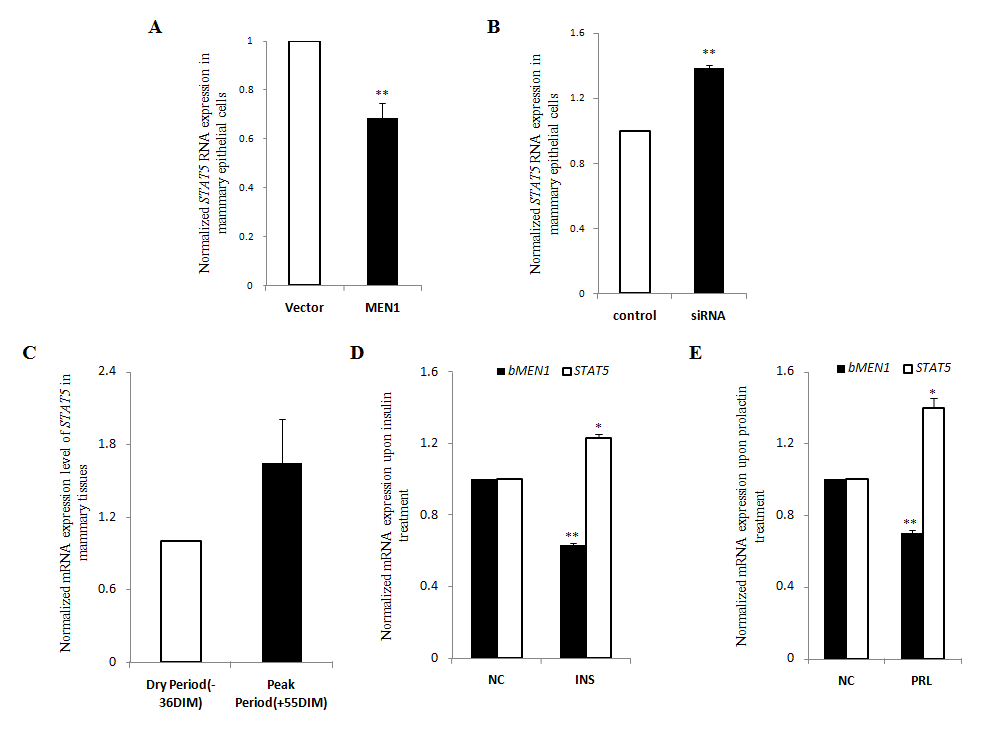
**

**Supplementary Figure S6. The expression of *STAT5* from the JAK2-STAT5 pathway was modulated upon the change of *MEN1*/Menin level in both mammary epithelial cells MAC-T and in tissue samples. (A) The expression of *STAT5* in MAC-T cells was significantly suppressed upon overexpression of *MEN1*/Menin. (B) The *STAT5* expression was significantly promoted in MAC-T cells upon reduced expression of *MEN1*/Menin. (C) The *STAT5* expression was higher in mammary gland tissues at dry period stage than peak milk stage, while *MEN1*/Menin expression showed opposite relative expression levels (Figure 4, supplementary Figure S5). (D) The *STAT5* expression was significantly promoted upon insulin (INS) treatment (5 μg/mL), while, *MEN1*/Menin expression showed significant suppression. (E) The *STAT5* expression was significantly promoted upon endocrine hormone prolactin (PRL) treatment (5 μg/mL), while, *MEN1*/Menin expression showed significant suppression. The data are shown as relative expression levels normalized to an internal control β-Actin. **P<*0.05, ** *P<*0.01.**

**Supplementary Table S1 Primer sequences of target genes studied for expression analysis in MAC-T cells and mammary gland tissues**

| Gene | Primer sequence (5'-3') | Product size |
| --- | --- | --- |
| *MEN1* | F: GATGGAGGTGGCATTTATGG | 256bp |
|  | R: GATGTGCTCATCCCGGTAGT |  |
| *Akt* | F: CCTGCCCTTCTACAACCAGG  R: GTCTTGGTCAGGTGGCGTAA | 328bp |
| *mTOR* | F: CGTCTCGCTTGTACTTTGGG  R: GCTGCTTGGAGATTCGTCTG | 243bp |
| *S6K1* | F: GTCAGGATGAGCTGGAGGAG  R: TCCCGGTATTTGCTCCTGTT | 234bp |
| *4E-BP1* | F: CTGGGGACTACAGCACCAC  R: AGGTGATTCTGCCTGGCTTC | 202bp |
| *STAT5* | F: GTCCCTTCCCGTGGTTGT  R: CGGCCTTGAATTTCATGTTG | 614bp |
| *CSNAS1*^1^ | F: AATCCATGCCCAACAGAAAG  R: TCAGAGCCAATGGGATTAGG | 189bp |
| *CSNAS2*^1^ | F: AGCTCTCCACCAGTGAGGAA  R: GCAAGGCGAATTTCTGGTAA | 150bp |
| *CSNB*^1^ | F: GTGAGGAACAGCAGCAAACA  R: TTTTGTGGGAGGCTGTTAGG | 115bp |
| *CSNK*^1^ | F: CCAGGAGCAAAACCAAGAAC  R: TGCAACTGGTTTCTGTTGGT | 148bp |
| *LGB*^1^ | F: CTTGTGCTGGACACCGACTA  R: TTGAGGGCTTTGTCGAATTT | 146bp |
| *LALBA*^1^ | F: AAAGACGACCAGAACCCTCA  R: GCTTTATGGGCCAACCAGTA | 143bp |
| *β-actin* | F: CCCAGCACAATGAAGATCAA  R: TAGAAGCATTTGCGGTGGAC | 180bp |

^1^ Primer pairs from previous reports (Zhou *et al*. 2008; Suzuki *et al*. 2015).

**Supplementary Table S2 The milk production information for cows that used for mammary tissue biopsy^1^**

| Lactation period | Cow No. | Parity | DIM^2^ | Milk yield(kg) | Fat percentage (%) | Protein percentage (%) | Somatic cell score | 305-milk yield (kg) |
| --- | --- | --- | --- | --- | --- | --- | --- | --- |
| Peak period | 1 | 1 | 50 | 30.4 | 2.94 | 2.52 | 0 | 7,583 |
|  | 2 | 3 | 56 | 34.2 | 3.4 | 2.81 | 2 | 7,644 |
|  | 3 | 2 | 59 | 38 | 5.25 | 2.79 | 1 | 9,773 |
| Dry period^3^ | 4 | 1 | 301 | 15.2 | 4.09 | 3.28 | 3 | 8,314 |
|  | 5 | 2 | 306 | 22.8 | 3.76 | 3.98 | 2 | 8,223 |
|  | 6 | 2 | 307 | 17.1 | 4.12 | 3.07 | 2 | 8,407 |

^1^ Milk was analyzed for protein, fat and somatic cell count (SCC) by the milk analysis laboratory of Livestock Improvement Corp (Dairy Cattle Research Center, Shandong Academy of Agricultural Sciences).

^2^ DIM, days in milking.

^3^ Dry period, the cows had been in their dry milking period for more than one month on the day of biopsy mammary gland tissues. Their milk production data were recorded on their lactation day as shown in the DIM column.
